# Supplementary material for: Cinnamomum migao H.W. Li Ethanol-Water Extract Suppresses IL-6 Production in Cardiac Fibroblasts: Mechanisms Elucidated via UPLC-Q-TOF-MS, Network Pharmacology, and Experimental Assays
Source: Curr Issues Mol Biol. 2025 Sep 26;47(10):798. doi: 10.3390/cimb47100798 (PMC12563024; doi:10.3390/cimb47100798)
Supplement: Supplementary file 1 [file cimb-47-00798-s001.zip › Table S1.pdf]

Table S1. The 173 chemical components of MG-EWE.

No.	MS information	CAS No.	Compound Name	Adducts	Formula	Mass Error (ppm)	Class
1	0.56_147.1120m/z	56-87-1	L-Lysine	M+H	C6H14N2O2	-5.483088818	Carboxylic acids and derivatives
2	0.56_175.1188m/z	7200-25-1	DL-Arginine	M+H	C6H14N4O2	-0.999967445	Carboxylic acids and derivatives
3	0.60_162.0774m/z	90-77-7	D-Glucosamine	M+H-H2O	C6H13NO5	7.388927364	Organooxygen compounds
4	0.69_527.1566m/z	597-12-6	Melezitose	M+Na	C18H32O16	-3.348511822	Organooxygen compounds
5	0.80_325.1128m/z	15548-39-7	2.alpha.-Mannobiose	M+H-H2O	C12H22O11	-0.31326136	Organooxygen compounds
6	0.85_268.1042m/z	58-61-7	Adenosine	M+H	C10H13N5O4	0.728328293	Purine nucleosides
7	0.87_182.0807m/z	60-18-4	L-Tyrosine	M+H	C9H11NO3	-2.744703296	Carboxylic acids and derivatives
8	0.87_252.1088m/z	958-09-8	2'-Deoxyadenosine	M+H	C10H13N5O3	-1.39907682	Purine nucleosides
9	0.89_137.0593m/z	10597-60-1	(3,4-Dihydroxyphenyl)ethanol	M+H-H2O	C8H10O3	-2.669858069	Phenols
10	0.89_152.0566m/z	73-40-5	Guanine	M+H	C5H5N5O	-0.546074888	Imidazopyrimidines

No.	MS information	CAS No.	Compound Name	Adducts	Formula	Mass Error (ppm)	Class
11	0.89_180.1015m/z	525-72-4	Salsolinol	M+H	C10H13NO2	-2.42250637	Tetrahydroisoquinolines
12	0.89_283.0914n	1818-71-9	Crotonoside	M+H, M+Na	C10H13N5O5	-1.115061688	Purine nucleosides
13	0.94_121.0643m/z	501-94-0	4-Hydroxyphenethyl alcohol	M+H-H2O	C8H10O2	-3.588138477	Phenols
14	1.03_132.1016m/z	73-32-5	L-Isoleucine	M+H	C6H13NO2	-2.171032155	Carboxylic acids and derivatives
15	1.23_162.0583m/z	21593-77-1	S-Allyl-L-Cysteine	M+H	C6H11NO2S	-0.393031921	Carboxylic acids and derivatives
16	1.45_137.1322m/z	546-79-2	Sabinene hydrate	M+H-H2O	C10H18O	-1.894793701	Prenol lipids
17	1.61_120.0805m/z	7568-93-6	2-Amino-1-phenylethanol	M+H-H2O	C8H11NO	-2.062371017	Organonitrogen compounds
18	1.61_165.0788n	63-91-2	L-Phenylalanine	M+H, M+Na	C9H11NO2	-0.853246358	Carboxylic acids and derivatives
19	1.85_334.1119m/z	2140-67-2	N,N-Dimethylguanosine	M+Na	C12H17N5O5	-0.892755216	Purine nucleosides
20	2.08_105.0700m/z	98-85-1	1-Phenylethanol	M+H-H2O	C8H10O	0.651519624	Benzene and substituted derivatives
21	2.15_136.1119m/z	99-97-8	N,N,4-Trimethylaniline	M+H	C9H13N	-1.555804383	Benzimidazoles

No.	MS information	CAS No.	Compound Name	Adducts	Formula	Mass Error (ppm)	Class
22	2.31_188.0706m/z	1204-06-4	3-Indoleacrylic acid	M+H	C11H9NO2	-0.065628632	Indoles and derivatives
23	2.37_272.1280m/z	5843-65-2	Higenamine	M+H	C16H17NO3	-0.433233597	Isoquinolines and derivatives
24	2.64_291.1012m/z	91216-95-4	.gamma.-Glutamyl-(S)-allyl-L-cysteine	M+H	C11H18N2O5S	0.816267858	Carboxylic acids and derivatives
25	2.76_325.0919m/z	93-39-0	Skimmin	M+H	C15H16O8	0.180365449	Coumarins and derivatives
26	2.91_149.0961m/z	1007-32-5	1-Phenyl-2-butanone	M+H	C10H12O	-0.121270056	Benzene and substituted derivatives
27	3.04_207.0652m/z	530-59-6	3,5-Dimethoxy-4-hydroxycinnamic acid	M+H-H2O	C11H12O5	0.030111755	Cinnamic acids and derivatives
28	3.45_168.0420n	61371-55-9	Griffonilide	M+H-H2O, M+H, M+Na	C8H8O4	-1.416208713	Benzofurans
29	3.45_314.1386m/z	5890-18-6	Laurolitsine	M+H	C18H19NO4	-0.22907398	Aporphines
30	3.56_133.1004m/z	536-60-7	Cuminyl alcohol	M+H-H2O	C10H14O	-5.182290175	Prenol lipids
31	3.65_290.0780n	154-23-4	(+)-Catechin	M+H, M+Na	C15H14O6	-3.609390824	Flavonoids
32	3.80_169.0758m/z	244-63-3	Norharmane	M+H	C11H8N2	-1.202989044	Indoles and derivatives
33	4.24_147.0434m/z	7400-08-0	p-Coumaric acid	M+H-H2O	C9H8O3	-4.077010774	Cinnamic acids and derivatives

No.	MS information	CAS No.	Compound Name	Adducts	Formula	Mass Error (ppm)	Class
34	4.27_325.1612m/z	32448-36-5	5-(Galactosylhydroxy)-L-lysine	M+H	C12H24N2O8	1.962690671	Carboxylic acids and derivatives
35	4.55_328.1540m/z	476-70-0	Boldine	M+H	C19H21NO4	-1.145981034	Aporphines
36	4.64_201.1639m/z	473-08-5	.alpha.-Cyperone	M+H-H2O	C15H22O	0.385289026	Prenol lipids
37	4.84_192.0423n	776-86-3	6-Hydroxy-7-methoxycoumarin	M+H, M+Na	C10H8O4	0.203689368	Coumarins and derivatives
38	4.93_184.1099n	473-72-3	cis-Pinonic acid	M+H-H2O, M+H, M+Na	C10H16O3	-0.373980378	Organooxygen compounds
39	4.95_194.0579n	1135-24-6	Ferulic acid	M+H-H2O, M+H	C10H10O4	-0.009136826	Cinnamic acids and derivatives
40	5.20_222.0540n	486-21-5	Isofraxidin	M+H, M+Na	C11H10O5	5.498734022	Coumarins and derivatives
41	6.02_153.1275m/z	464-49-3	(+)-Camphor	M+H	C10H16O	0.442333703	Prenol lipids
42	6.09_151.1114m/z	99-49-0	Carvone	M+H	C10H14O	-2.185456562	Prenol lipids
43	6.29_125.0957m/z	698-76-0	.delta.-Octalactone	M+H-H2O	C8H14O2	-3.076972076	Lactones
44	6.64_209.1524m/z	2630-39-9	Methyl dihydrojasmonate	M+H-H2O	C13H22O3	-5.513698408	Fatty Acyls
45	6.89_168.1150n	14087-70-8	(1R)-Chrysanthemolactone	M+H-H2O, M+H, M+Na	C10H16O2	-0.470522389	Lactones

No.	MS information	CAS No.	Compound Name	Adducts	Formula	Mass Error (ppm)	Class
46	7.00_217.1587m/z	6754-20-7	Polygodial	M+H-H2O	C15H22O2	0.027234431	Organic oxides
47	7.04_415.3187m/z	949004-12-0	(25S)-7-Dafachronic acid	M+H	C27H42O3	-4.80956548	Steroids and steroid derivatives
48	7.31_235.1691m/z	609870	Valerenic acid	M+H	C15H22O2	-0.500856513	Prenol lipids
49	7.58_135.1167m/z	19894-97-4	(-)-Myrtenol	M+H-H2O	C10H16O	-0.9769607	Prenol lipids
50	8.12_109.0638m/z	108-39-4	m-Cresol	M+H	C7H8O	-9.634082207	Phenols
51	8.12_151.1119m/z	6485-40-1	L-Carvone	M+H	C10H14O	0.979568031	Prenol lipids
52	8.54_133.1009m/z	2111-75-3	Perillaldehyde	M+H-H2O	C10H14O	-1.820325754	Prenol lipids
53	8.57_105.0697m/z	587-03-1	3-Methylbenzyl alcohol	M+H-H2O	C8H10O	-1.693570483	Benzene and substituted derivatives
54	8.70_236.1777n	4871-97-0	Curcumol	M+H-H2O, M+H, M+Na	C15H24O2	0.339165476	Prenol lipids
55	9.69_205.1951m/z	77-53-2	Cedrol	M+H-H2O	C15H26O	-0.028661284	Prenol lipids
56	9.85_137.1323m/z	470-82-6	Eucalyptol	M+H-H2O	C10H18O	-1.070258402	Oxanes
57	10.28_136.1251n	3387-41-5	Sabinene	M+H, M+Na	C10H16	-1.015110062	Prenol lipids
58	10.35_192.1151n	63038-10-8	Senkyunolide A	M+H-H2O, M+H, M+Na	C12H16O2	0.257607503	Isobenzofurans
59	10.37_164.1199n	488-10-8	cis-Jasmone	M+H-H2O, M+H	C11H16O	-1.471585294	Organooxygen compounds
60	10.53_145.1007m/z	4160-52-5	1-(p-Tolyl)butan-1-one	M+H-H2O	C11H14O	-2.791168209	Organooxygen compounds

No.	MS information	CAS No.	Compound Name	Adducts	Formula	Mass Error (ppm)	Class
61	11.14_194.1305n	6415-59-4	Sedanolide	M+H-H2O, M+H, M+Na	C12H18O2	-1.125606095	Isobenzofurans
62	11.14_205.1952m/z	489-41-8	(-)-Globulol	M+H-H2O	C15H26O	0.758180669	Prenol lipids
63	11.21_190.0993n	81944-09-4	Z-Ligustilide	M+H-H2O, M+H, M+Na	C12H14O2	-0.569987928	Isobenzofurans
64	11.43_196.1463n	24738-48-5	Dodeca-2(E),4(E)-dienoic acid	M+H-H2O, M+H, M+Na	C12H20O2	-0.238378025	#N/A
65	11.95_201.1638m/z	4674-50-4	Nootkatone	M+H-H2O	C15H22O	0.034547057	Prenol lipids
66	12.04_218.1672n	471-05-6	Zerumbone	M+H, M+Na	C15H22O	0.407383006	Prenol lipids
67	12.38_263.2384m/z	506-21-8	Linoelaidic acid	M+H-H2O	C18H32O2	5.283597049	Fatty Acyls
68	12.46_402.2393n	81-23-2	Dehydrocholic acid	M+H-H2O, M+	C24H34O5	-3.227691096	Steroids and steroid derivatives
69	12.49_278.2246n	16833-54-8	Pinolenic acid	M+H, M+Na	C18H30O2	-0.021682776	Fatty Acyls
70	12.49_341.2070m/z	141110-17-0	18-Hydroxy-5Z,8Z,11Z,14Z,16E-eicosapentaenoic acid	M+Na	C20H30O3	-5.510076425	Fatty Acyls

No.	MS information	CAS No.	Compound Name	Adducts	Formula	Mass Error (ppm)	Class
71	12.62_175.1479m/z	127-41-3	.alpha.-Ionone	M+H-H2O	C13H20O	-1.305336227	Prenol lipids
72	12.62_203.1794m/z	19317-11-4	Farnesal	M+H-H2O	C15H24O	0.071894923	Prenol lipids
73	13.10_278.1517n	84-74-2	Dibutyl phthalate	M+H, M+Na	C16H22O4	-0.503787207	Benzene and substituted derivatives
74	13.12_149.0231m/z	88-99-3	1,2-Benzenedicarboxylic acid	M+H-H2O	C8H6O4	-1.129400512	Benzene and substituted derivatives
75	13.21_205.1952m/z	515-69-5	.alpha.-Bisabolol	M+H-H2O	C15H26O	0.53846505	Prenol lipids
76	13.23_381.2057m/z	88182-33-6	Levistolide A	M+H	C24H28O4	-0.813875865	Dihydrofurans
77	13.47_205.1951m/z	7212-44-4	(cis+trans)-Nerodilol	M+H-H2O	C15H26O	0.249168202	Prenol lipids
78	13.89_323.2820n	68171-52-8	Linoleoyl ethanolamide	M+H, M+Na	C20H37NO2	-1.301960794	Organonitrogen compounds
79	14.17_431.3143m/z	467-55-0	Hecogenin	M+H	C27H42O4	-2.898022575	Prenol lipids
80	14.94_354.2763n	2277-28-3	1-Monolinoleoyl-rac-glycerol	M+H-H2O, M+H, M+Na	C21H38O4	-1.865969798	Fatty Acyls

No.	MS information	CAS No.	Compound Name	Adducts	Formula	Mass Error (ppm)	Class
81	15.71_280.2401n	544-71-8	9E,11E-Octadecadienoic acid	M+H-H2O, M+H, M+Na	C18H32O2	-0.31486238	Fatty Acyls
82	16.03_356.2923n	111-03-5	Monoolein	M+H-H2O, M+H, M+Na	C21H40O4	-0.977215519	Glycerolipids
83	16.74_282.2558n	506-17-2	cis-Vaccenic acid	M+H-H2O, M+H, M+Na	C18H34O2	-0.189888212	Fatty Acyls
84	18.01_337.3343n	112-84-5	Erucamide	M+H, M+Na	C22H43NO	-0.539188188	Fatty Acyls
85	18.91_137.1322m/z	53369-17-8	(-)-trans-Myrtanol	M+H-H2O	C10H18O	-1.584343814	Prenol lipids
86	21.55_337.2736m/z	3443-82-1	2-Linoleoylglycerol	M+H-H2O	C21H38O4	-0.238861843	Fatty Acyls
87	6.51_362.1724n	29388-59-8	(-)-Secoisolariciresinol	M-H, M+FA-H	C20H26O6	-1.402976163	Dibenzylbutane lignans
88	6.29_239.0925m/z	86879-39-2	2-Furanpropanoic acid, 3-carboxy-4-methyl-5-propyl-	M-H	C12H16O5	0.060402427	Fatty Acyls
89	3.12_151.0410m/z	673-22-3	2-Hydroxy-4-methoxybenzaldehyde	M-H	C8H8O3	6.021587066	Phenols

No.	MS information	CAS No.	Compound Name	Adducts	Formula	Mass Error (ppm)	Class
90	2.90_175.0611m/z	3237-44-3	2-Isopropylmalic acid	M-H	C7H12O5	-0.421160053	Fatty Acyls
91	5.17_173.0821m/z	32806-62-5	2-Propylglutaric acid	M-H	C8H14O4	0.68455148	Fatty Acyls
92	5.91_179.0714m/z	10516-71-9	3-(3-Methoxyphenyl) propionic acid	M-H	C10H12O3	0.411810165	Phenylpropanoic acids
93	3.30_159.0656m/z	4839-46-7	3,3-Dimethylglutaric acid	M-H	C7H12O4	-4.374964432	Fatty Acyls
94	9.89_307.1907m/z	1035557-09-5	3-Cyclopentene-1-octanoic acid, 2-(3-hydroxy-1-penten-1-yl)-5-oxo-	M-H	C18H28O4	-2.516266467	#N/A
95	0.99_161.0454m/z	503-49-1	3-Hydroxy-3-methylglutaric acid	M-H	C6H10O5	-1.010379581	Fatty Acyls
96	1.08_216.0999n	114212-45-2	3-Oxo-1,8-octanedicarboxylic acid	M-H, M+FA-H	C10H16O5	0.782230461	Keto acids and derivatives
97	9.66_163.0763m/z	4593-90-2	3-Phenylbutyric acid	M-H	C10H12O2	-0.988499863	Phenylpropanoic acids

No.	MS information	CAS No.	Compound Name	Adducts	Formula	Mass Error (ppm)	Class
98	6.18_515.1192m/z	57378-72-0	4,5-Dicaffeoylquinic acid	M-H	C25H24O12	-0.64443581	Organooxygen compounds
99	2.54_181.0142m/z	618-83-7	5-Hydroxyisophthalic acid	M-H	C8H6O5	-0.018335372	Benzene and substituted derivatives
100	11.28_313.2380m/z	263399-34-4	9,10-Dihydroxy-12Z-octadecenoic acid	M-H	C18H34O4	-1.324050199	Fatty Acyls
101	12.85_293.2119m/z	54232-58-5	9-Oxo-10(E),12(E)-octadecadienoic acid	M-H	C18H30O3	-0.941393378	Fatty Acyls
102	1.37_242.0904n	50-89-5	Thymidine	M-H, M+FA-H	C10H14N2O5	0.564013622	Pyrimidine nucleosides
103	0.59_225.0617m/z	59-23-4	D-(+)-Galactose	M+FA-H	C6H12O6	0.609037624	Organooxygen compounds
104	15.72_280.2399n	60-33-3	Linoleic acid	M-H, M+FA-H	C18H32O2	-1.31073411	Fatty Acyls
105	2.79_191.0561m/z	77-95-2	(-)-Quinic acid	M-H	C7H12O6	-0.093572721	Organooxygen compounds
106	3.88_387.1144m/z	99-20-7	D-(+)-Trehalose	M+FA-H	C12H22O11	-0.069804185	Organooxygen compounds

No.	MS information	CAS No.	Compound Name	Adducts	Formula	Mass Error (ppm)	Class
107	6.04_137.0242m/z	99-96-7	4-Hydroxybenzoic acid	M-H	C7H6O3	-1.66593423	Benzene and substituted derivatives
108	3.48_253.0714m/z	102-37-4	Ethyl caffeate	M+FA-H	C11H12O4	-1.90886696	Cinnamic acids and derivatives
109	2.25_203.0826m/z	153-94-6	D-(+)-Tryptophan	M-H	C11H12N2O2	-0.029056166	Indoles and derivatives
110	3.08_177.0193m/z	305-01-1	Esculetin	M-H	C9H6O4	-0.312357863	Coumarins and derivatives
111	5.77_177.0555m/z	458-36-6	Coniferyl aldehyde	M-H	C10H10O3	-1.11337764	Phenols
112	2.11_163.0408m/z	501-98-4	p-Coumaric acid	M-H	C9H8O3	4.25299816	Cinnamic acids and derivatives
113	5.95_447.0928m/z	522-12-3	Quercitrin	M-H	C21H20O11	-1.055231228	Flavonoids
114	5.57_327.1448m/z	1034-01-1	Octyl gallate	M+FA-H	C15H22O5	-0.426430334	Benzene and substituted derivatives
115	5.48_245.0928m/z	1218-34-4	N-Acetyl-DL-tryptophan	M-H	C13H14N2O3	-1.363986915	Carboxylic acids and derivatives
116	5.35_195.0660m/z	2478-38-8	1-(4-Hydroxy-3,5-dimethoxyphenyl)ethanone	M-H	C10H12O4	-1.370901931	Organooxygen compounds

No.	MS information	CAS No.	Compound Name	Adducts	Formula	Mass Error (ppm)	Class
117	3.57_415.1037m/z	3681-99-0	Puerarin	M-H	C21H20O9	0.548911552	Coumarins and derivatives
118	0.99_323.0992m/z	5989-81-1	α-Lactose	M-H2O-H	C12H22O11	2.301712769	#N/A
119	6.47_435.1294m/z	7061-54-3	Phloridzin	M-H	C21H24O10	-0.592916989	Flavonoids
120	4.02_511.1442m/z	11027-63-7	Agnuside	M+FA-H	C22H26O11	-3.248358121	Prenol lipids
121	5.68_264.0994n	20516-23-8	Peucedanol	M-H, M+FA-H	C14H16O5	-1.566268058	Coumarins and derivatives
122	10.34_293.1755m/z	23513-14-6	[6]-Gingerol	M-H	C17H26O4	-1.285647532	Phenols
123	5.10_787.2687m/z	39432-56-9	Eleutheroside E	M+FA-H	C34H46O18	2.843485656	Lignan glycosides
124	11.66_740.4356n	41059-79-4	Timosaponin A-III	M-H, M+FA-H	C39H64O13	1.225136697	Steroids and steroid derivatives
125	5.95_329.1605m/z	71939-50-9	Dihydroartemisinin	M+FA-H	C15H24O5	-0.395285649	Prenol lipids
126	3.79_341.1621n	104112-82-5	Phellodendrine	M-H, M+FA-H	C20H23NO4	-1.713673165	Protoberberine alkaloids and derivatives
127	1.84_583.1290m/z	241125-81-5	Sibiricoxanthone B	M+FA-H	C24H26O14	-2.779410872	Benzopyrans
128	0.86_188.0571m/z	1430871	N-Acetyl-DL-glutamic acid	M-H	C7H11NO5	3.407742986	Carboxylic acids and derivatives
129	6.29_187.0973m/z	123-99-9	Azelaic acid	M-H	C9H16O4	-1.498979017	Fatty Acyls

No.	MS information	CAS No.	Compound Name	Adducts	Formula	Mass Error (ppm)	Class
130	4.85_227.1284m/z	5703-15-1	Butanedioic acid, 2-(4,4-dimethyl-2-methylenepentyl)-	M-H	C12H20O4	-2.211711944	Fatty Acyls
131	3.19_179.0349m/z	331-39-5	Caffeic acid	M-H	C9H8O4	-0.290577471	Cinnamic acids and derivatives
132	0.83_191.0195m/z	77-92-9	Citric acid	M-H	C6H8O7	-1.094080183	Carboxylic acids and derivatives
133	0.59_504.1691n	512-69-6	D-(+)-Raffinose	M-H, M+FA-H	C18H32O16	0.134899457	Organooxygen compounds
134	0.63_165.0405m/z	488-30-2	D-Arabinonic acid	M-H	C5H10O6	0.178820559	Carboxylic acids and derivatives
135	0.61_195.0509m/z	526-95-4	D-Gluconic acid	M-H	C6H12O7	-0.47148303	Hydroxy acids and derivatives
136	0.68_133.0137m/z	6915-15-7	DL-Malic acid	M-H	C4H6O5	-3.760984734	Carboxylic acids and derivatives
137	0.86_128.0351m/z	4042-36-8	D-Pyroglutamic acid	M-H	C5H7NO3	-1.573524031	Carboxylic acids and derivatives
138	3.75_225.0767m/z	3943-80-4	Ethyl syringate	M-H	C11H14O5	-0.815313692	Benzene and substituted derivatives
139	1.69_131.0349m/z	601-75-2	Ethylmalonic acid	M-H	C5H8O4	-0.76105475	Fatty Acyls

No.	MS information	CAS No.	Compound Name	Adducts	Formula	Mass Error (ppm)	Class
140	5.21_221.0460m/z	486-28-2	Fraxinol	M-H	C11H10O5	2.055526208	Coumarins and derivatives
141	1.84_153.0194m/z	490-79-9	Gentisic acid	M-H	C7H6O4	0.593947178	Benzene and substituted derivatives
142	11.71_285.2072m/z	505-54-4	Hexadecanedioic acid	M-H	C16H30O4	0.352045114	Fatty Acyls
143	0.94_117.0186m/z	516-05-2	Methylmalonic acid	M-H	C4H6O4	-5.920450459	Carboxylic acids and derivatives
144	0.63_209.0307m/z	526-99-8	Mucic acid	M-H	C6H10O8	2.089183002	Carboxylic acids and derivatives
145	5.05_206.0821m/z	2018-61-3	N-Acetyl-L-phenylalanine	M-H	C11H13NO3	-0.651657422	Carboxylic acids and derivatives
146	3.43_312.1244m/z	23599-69-1	Norisoboldine	M-H	C18H19NO4	1.005263511	Aporphines
147	8.58_305.1016m/z	31858-65-8	O-Desmethylmycophenolic acid	M-H	C16H18O6	-4.738622313	#N/A
148	1.78_218.1036m/z	599-54-2	Pantothenic acid	M-H	C9H17NO5	0.989381089	Organooxygen compounds
149	9.52_165.0920m/z	98-29-3	p-tert-Butylcatechol	M-H	C10H14O2	-0.755293616	Benzene and substituted derivatives
150	1.12_173.0455m/z	138-59-0	Shikimic acid	M-H	C7H10O5	-0.51481725	Organooxygen compounds

No.	MS information	CAS No.	Compound Name	Adducts	Formula	Mass Error (ppm)	Class
151	8.76_227.1285m/z	6402-36-4	trans-Traumatic acid	M-H	C12H20O4	-1.573331289	Fatty Acyls
152	8.35_215.1291m/z	1852-04-6	Undecanedioic acid	M-H	C11H20O4	0.864582251	Fatty Acyls
153	14.94_271.2276m/z	764-67-0	2-Hydroxypalmitic acid	M-H	C16H32O3	-0.95812513	Fatty Acyls
154	3.39_167.0346m/z	28026-96-2	3,5-Dihydroxy-4-methylbenzoic acid	M-H	C8H8O4	-2.021794021	Benzene and substituted derivatives
155	11.82_294.2190n	89886-42-0	9S-Hydroxy-10E,12Z,15Z-octadecatrienoic acid	M-H2O-H, M-H	C18H30O3	-1.610105773	Fatty Acyls
156	12.49_295.2275m/z	948310-68-7	12(13)-Epoxy-9Z-octadecenoic acid	M-H	C18H32O3	-1.21342989	Fatty Acyls
157	11.16_313.2378m/z	263399-35-5	12,13-Dihydroxy-9Z-octadecenoic acid	M-H	C18H34O4	-1.930533001	Fatty Acyls
158	11.91_315.2537m/z	112-39-0	Methyl hexadecanoate	M+FA-H	C17H34O2	-1.31409422	Fatty Acyls

No.	MS information	CAS No.	Compound Name	Adducts	Formula	Mass Error (ppm)	Class
159	2.47_137.0242m/z	139-85-5	3,4-Dihydroxybenzaldehyde	M-H	C7H6O3	-1.745542134	Organooxygen compounds
160	4.02_172.0978m/z	7682-16-8	2-(Acetylamino)hexanoic acid	M-H	C8H15NO3	-0.416599622	Carboxylic acids and derivatives
161	5.82_515.1194m/z	30964-13-7	Cynarin	M-H	C25H24O12	-0.261394942	Organooxygen compounds
162	1.53_164.0715m/z	150-30-1	DL-Phenylalanine	M-H	C9H11NO2	-1.194366425	Carboxylic acids and derivatives
163	4.72_195.0668m/z	1135-23-5	Hydroferulic acid	M-H	C10H12O4	2.595540088	Phenylpropanoic acids
164	4.83_191.0350m/z	92-61-5	Scopoletin	M-H	C10H8O4	-0.014432408	Coumarins and derivatives
165	4.72_165.0558m/z	828-01-3	DL-3-Phenyllactic acid	M-H	C9H10O3	0.780203519	Phenylpropanoic acids
166	4.63_183.0661m/z	778649-18-6	3-Furancarboxylic acid, tetrahydro-4-methylene-5-oxo-2-propyl-, (2R,3S)-rel-	M-H	C9H12O4	-0.806813023	#N/A

No.	MS information	CAS No.	Compound Name	Adducts	Formula	Mass Error (ppm)	Class
167	4.43_682.2502n	63902-38-5	Pinoresinol diglucoside	M-H, M+FA-H	C32H42O16	4.296316174	Lignan glycosides
168	4.72_407.1349m/z	80358-06-1	Tinnevellin glucoside	M-H	C20H24O9	0.292296715	Organooxygen compounds
169	4.38_159.0662m/z	681-57-2	2,2-Dimethylglutaric acid	M-H	C7H12O4	-0.774681695	Fatty Acyls
170	4.24_163.0400m/z	614-60-8	trans-2-Hydroxycinnamic acid	M-H	C9H8O3	-0.588697622	Cinnamic acids and derivatives
171	4.18_390.0926n	58130-91-9	4',5,7-Trihydroxy 3,3',6,8-tetramethoxyflavone	M-H, M+FA-H	C19H18O9	-6.466132886	Flavonoids
172	4.13_151.0399m/z	621-59-0	3-Hydroxy-4-methoxybenzaldehyde	M-H	C8H8O3	-1.150076052	Phenols
173	4.83_549.1982m/z	33464-71-0	Tracheloside	M-H	C27H34O12	0.800160651	Lignan glycosides
